# Editorial: Advances in the research of diabetic retinopathy, volume III

**DOI:** 10.3389/fendo.2025.1628562

**Published:** 2025-05-28

**Authors:** Mohd Imtiaz Nawaz, Sara Rezzola

**Affiliations:** ^1^ Department of Ophthalmology, College of Medicine, King Saud University, Riyadh, Saudi Arabia; ^2^ Dr. Nasser Al-Rashid Research Chair in Ophthalmology, Abdulaziz University Hospital, Riyadh, Saudi Arabia; ^3^ Department of Molecular and Translational Medicine, University of Brescia, Brescia, Italy

**Keywords:** diabetic retinopathy (DR), biomarkers, Mendelian randomization (MR) analysis, optical coherence tomography angiography (OCTA), predictive risk model, therapeutic targets

Diabetic Retinopathy (DR) is a major neurovascular disorder of the retina that causes serious vision loss in the working-age population worldwide. The long-term effects of diabetes mellitus initiate multiple dysregulated metabolic pathways in the retina. Subsequently, the onset and progression of DR engage the simultaneous involvements of early neurodegenerative events, oxidative stress, inflammation, and angiogenesis, finally culminating in irreversible fibrotic changes (1, 2).

Surprisingly, the appearance of clinical manifestation of DR takes several years, making the early detection and the diagnosis difficult for clinicians. In addition, current treatment interventions for advanced proliferative DR (PDR) – including vitreous hemorrhage, macular edema, formation of the epiretinal membrane, and retinal neovascularization – are also limited. Among available treatment strategies, vitrectomy and laser photocoagulation represent the traditional approach for managing PDR-associated vision-threatening conditions (3). Moreover, breakthroughs in understanding the starring role of major angiogenic mediator Vascular Endothelial Growth Factor (VEGF) in the initiation and progression of DR-associated pathogenesis have led to the development of anti-VEGF drugs (e.g., pegaptanib, bevacizumab, ranibizumab, and aflibercept/VEGF Trap-Eye) as therapies for PDR management (1, 3). Indeed, VEGF is considered a key players in the pathophysiological mechanisms underlying PDR. Unfortunately, each treatment strategy has its limitations. Likewise, vitrectomy and laser photocoagulation are invasive and destructive procedures. Similarly, anti-VEGF drugs or corticosteroids are limited by short duration of action, a poor response in a significant percentage of patients, and the presence of adverse side effects (4, 5). Additionally, interventions for early-stage DR are also inadequate.

Thus, thinking outside the box to improve our knowledge of DR pathogenesis and corresponding management strategies, future research should (i) re-evaluate existing and/or explore new molecular and pathophysiological pathways, possibly at different stages of the disease; (ii) develop innovative approaches for early detection and cost-effective strategies, with the aim to manage DR at its earliest stage, rather than waiting for the onset of non-manageable forms of vision-threatening lesions.

Given the overwhelming success of the previous two editions of the Research Topic *Advances in the Research of Diabetic Retinopathy* (6, 7), and the ongoing research advancements in the field, we launched *Volume III* of the edition. The goal was to compile a dossier highlighting new and exciting experimental evidence to advance the understanding of retinal vascular damage and its underlying cellular and molecular mechanisms.

In response to this purpose, *Volume III* attracted numerous exciting original articles and reviews. Notably, the volume includes several contributions discussing emerging therapeutic strategies for DR managing.

In this frame, a significant work by Sabeti et al. developed a sensitive functional test to investigate the effects of early-stage DR on visual function across the central and peripheral retina, using two multifocal pupillographic objective perimetry (mfPOP) stimulus methods. The results of this study demonstrate the utility and advantage of mfPOP in detecting early changes in visual function in type 2 diabetes (T2D) patients.

Other methods, including meta-analysis, multivariable Mendelian randomization (MR) analysis, application of advanced machine learning, and cross-sectional retrospective investigations, represent alternative strategies for predicting correlations between molecular variables and the degree of DR in T2D patients.

Using meta-analysis, Jiang et al. and Ouyang et al. demonstrated significant associations between fibroblast growth factor 21 (FGF21) or thyroid dysfunction and DR, respectively. Fully characterizing their role may significantly contribute to understanding the pathogenesis of DR.

In a study by Wang et al., the molecular complexity of PDR was unveiled employing single-cell RNA sequencing (scRNA-seq), AlphaFold 2, and machine learning methods. This study deepened our understanding of oxidative stress-related genes - ALKBH1, PSIP1, and ATP13A2 – which could serve as biomarkers and enhance both diagnostic and therapeutic strategies for PDR.

Similarly, bioinformatic analysis of a public database may offer another tool to gain novel insight into potential biomarkers that may play a role in DR pathogenesis. For instance, Liu et al. identified three genes (OSER1, HIPK2, and DDRGK1) as potential biomarkers in DR pathogenesis. The value of these types of studies lies also in their potential to assist pharmaceutical companies in rapidly identifying biomarker-targeting strategies for DR management.

Remarkably, researchers have also been working tirelessly to identify correlations between novel molecular variables and DR progression. In this context, a cross-sectional study led by Ding et al., demonstrated that the HALP score obtained by measuring the serum levels of hemoglobin, albumin, lymphocyte, and platelet, has an L-shaped correlation with the risk of DR. In addition, based on the well-known roles of Aspartate aminotransferase (AST) and Alanine aminotransferase (ALT) in metabolic and inflammatory processes, a study reported a positive association between the AST/ALT ratio and presence of DR, suggesting that clinical scores may contribute to early detection, risk stratification, and timely interventions in DR patients (Li et al.).

Even though the retina is a highly metabolically active tissue, there is still a significant gap in study about the relationship between metabolites and the initiation and progression of DR. In an attempt to fill some gap in this area, Li et al. utilized open-access genome-wide association studies (GWAS) database to identify potential metabolites involved in the pathogenesis of DR. This Mendelian randomization study suggests that 9 metabolites were negatively associated with the risk of DR, while two metabolites, i.e., 5-hydroxymethyl-2-furoylcarnitine and the glutamate-to-alanine ratio, may be associated with an increased risk of DR.

A real-world study by Guo et al. investigated arginine pathway metabolites by utilizing plasma and aqueous humor samples from PDR patients. This study showed that among the arginine pathway metabolites (L-arginine, asymmetric dimethylarginine (ADMA), L-ornithine, and L-citrulline) ADMA levels were elevated both in plasma and aqueous humor compared to the control patients, positively correlating with the severity of fibrovascular proliferation in PDR. These findings indicate that ADMA could represent a risk factor for severe PDR. Thus, future research assessing the impact of modulating ADMA levels on PDR development is warranted.

Recent advances in scanning optical coherence tomography angiography (OCTA), a non-invasive imaging technique, suggests its potential as valuable clinical tool for detecting early diabetes-induced retinal and choroidal microvasculature changes in DR patients. In this frame, a review article by Zhang et al. discussed in detail the OCTA’s imaging principles, its applications in detecting DR lesions, and its diagnostic advantages over fundus fluorescein angiography.

Further supporting the clinical usefulness of OCTA, Jing et al. demonstrated that a decrease in the choroidal vascularity index (CVI) over the course of diabetes correlates with visual impairment, indicating that CVI could serve as a reliable imaging biomarker to monitor the progression of DR. Similarly, He et al. showed a decrease in retinal and choroidal thickness as well as vessel density (VD), with a strong correlation between tissue thickness and VD in patients with non-PDR. In conclusion, these and other similar studies highlight the significance of OCTA-derived parameters as a predictive indicator of the severity of DR, providing a promising strategy for the early diagnosis and intervention of DR.

As stated above, several therapeutic approaches have been developed over the years to manage DR. In this context, Reddy et al. critically reviewed the benefits and limitations of existing DR management strategies. Among emerging treatments, cell-based therapies, for instance transplantation or cell engineering, are gaining interest as new approaches. For example, Pelusi et al. evaluated the effects of mesenchymal stromal cells (MSCs) derived from human amniotic fluids (hAFSCs) and recombinant human nerve growth factor (rhNGF), delivered via bioengineered human corneal lenticule (hCL) in an *ex vivo* porcine neuroretinal explant model exposed to high glucose (HG). Their findings suggest that hAFSCs and rhNGF can modulate key molecular mechanisms involved in DR, and that bioengineered hCLs may represent a promising platform for ocular drug delivery. In addition, the use of porcine neuroretinal explants treated with HG could be a useful model to reproduce *ex vivo* DR pathophysiology.

Finally, several studies have demonstrated that changes in the environment around or lifestyle modifications can alter circadian rhythm that could, in turn, accelerate diabetes-related complications. Accordingly, data from a recent study by Wang et al. suggests that maintaining a more regular sleep-activity cycle might mitigate the risk of DR development. Thus, beyond conventional clinical strategies, promoting a circadian rhythm stability and increasing diurnal activity may effectively mitigate the risk of progression of DR and diabetes-associated complications at large in a non-pharmacological manner.

In conclusion, *Volume III* of this Research Topic offers new insights and novel valuable data toward understanding the early retinal vascular damage and the pathological mechanism underlying the onset and progression of DR. The use of a high-end predictive models could provide a wealth of knowledge into the early assessment and pathological grading of DR. Furthermore, contributions focused on the identification and characterization of novel potential biomarkers could open new therapeutic avenues for the management of DR.

The editors of this Research Topic believe that the collection of articles presented in this volume represents a valuable addition to the existing body of clinical and basic research on DR. However, as scientific knowledge evolves without boundaries, the editors encourage more interdisciplinary research aimed at improving early diagnosis of the disease and developing of cost-effective treatment strategies for the management of DR.
